# ADP-ribose contributions to genome stability and PARP enzyme trapping on sites of DNA damage; paradigm shifts for a coming-of-age modification

**DOI:** 10.1016/j.jbc.2023.105397

**Published:** 2023-10-28

**Authors:** Élise Rouleau-Turcotte, John M. Pascal

**Affiliations:** Department of Biochemistry and Molecular Medicine, Université de Montréal, Montréal, Quebec, Canada

**Keywords:** ADP-ribose, ADP-ribosyltransferase, poly(ADP-ribose), DNA damage, DNA strand breaks, PARP enzymes, PARP trapping, PARP inhibitors, allostery, chromatin, poly(ADP-ribose) glycohydrolases, PARG, ARH3, protein–nucleic acid interactions

## Abstract

ADP-ribose is a versatile modification that plays a critical role in diverse cellular processes. The addition of this modification is catalyzed by ADP-ribosyltransferases, among which notable poly(ADP-ribose) polymerase (PARP) enzymes are intimately involved in the maintenance of genome integrity. The role of ADP-ribose modifications during DNA damage repair is of significant interest for the proper development of PARP inhibitors targeted toward the treatment of diseases caused by genomic instability. More specifically, inhibitors promoting PARP persistence on DNA lesions, termed PARP “trapping,” is considered a desirable characteristic. In this review, we discuss key classes of proteins involved in ADP-ribose signaling (writers, readers, and erasers) with a focus on those involved in the maintenance of genome integrity. An overview of factors that modulate PARP1 and PARP2 persistence at sites of DNA lesions is also discussed. Finally, we clarify aspects of the PARP trapping model in light of recent studies that characterize the kinetics of PARP1 and PARP2 recruitment at sites of lesions. These findings suggest that PARP trapping could be considered as the continuous recruitment of PARP molecules to sites of lesions, rather than the physical stalling of molecules. Recent studies and novel research tools have elevated the level of understanding of ADP-ribosylation, marking a coming-of-age for this interesting modification.

## ADP-ribose modifications and genome stability

DNA carries the necessary information for many processes within the cell and maintaining its stability is of critical importance to ensure cell viability. Genome instability can arise from endogenous causes, such as normal genome transactions (replication, transcription, recombination), but also from exogenous causes, like external genome damaging agents (1). The sheer number of lesions each human cell experiences daily (approximately 70,000 lesions) (2) highlights the heavy demand put on genome maintenance mechanisms. As such, a variety of DNA repair pathways exist to tackle the diversity and abundance of lesions, with many of these pathways carrying overlapping functions (1). DNA repair pathways rely on the interplay between enzymes and posttranslational modifications (PTMs) (phosphorylation, ubiquitylation, SUMOylation, etc) to proceed with success (3).

**Figure 1:**
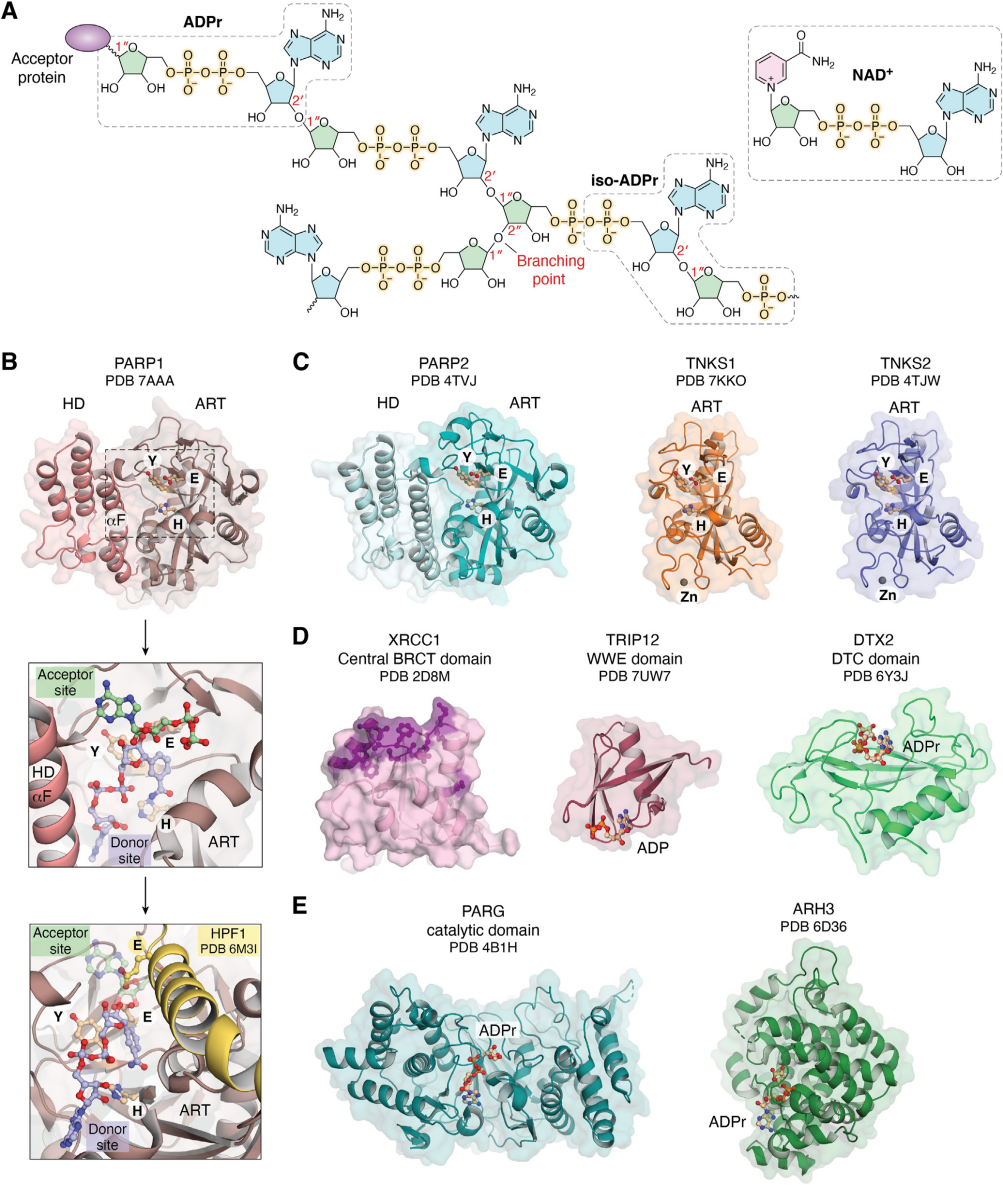
**The ADPr/PAR modification and proteins involved in its synthesis, turnover, and signaling.***A*, schematic representation of a PAR chain composed of (2′-1″) ribose–ribose glycosidic bonds (chain elongation) and occasionally a (2″-1″) ribose–ribose bond (branching point). The modification is attached to target proteins *via* the anomeric C1″ atom of the first ADPr moiety. The ADPr moiety represents the repeated unit of a PAR chain. *Iso*-ADPr is highlighted as well as NAD^+^, the substrate from which ADPr originates. *B*, the catalytic domain of PARP1 (*pink*, PDB 7AAA (111)) with the conserved HYE catalytic triad represented in *sticks* in the ADP-ribosyltransferase (ART) domain. A close-up view of the PARP1 catalytic pocket is represented in the *upper inset*. The substrate analog BAD (*light blue carbons*, 6BHV (52)) is depicted in the donor site, while carba-NAD^+^ (*green carbons*, 1A26 (152)) is represented in the acceptor site. Histone PARylation factor 1 (*yellow*, 6M3I (38)) binding to PARP1 (*lower inset*) occludes the acceptor site and results in the insertion of residue E284 to drive Ser-linked modification. *C*, the catalytic domain of PARP2 (*teal*, 4TVJ (153)), TNKS1 (*orange*, 7KKO (106)), and TNKS2 (*violet*, 4TJW (154)). Both PARP1 and PARP2 possess an autoinhibitory subdomain, the helical domain (HD), that regulates their catalytic activity by restricting access to NAD^+^. TNKS1 and TNKS2 do not possess an HD regulatory domain. In general, MARylation enzymes lack the glutamate in the HYE motif, or lack a functional acceptor site, but otherwise have the same overall ART domain. *D*, diverse domains that “read” the PAR modification. The central BRCT domain of XRCC1 (*pink*, 2D8M) with the expected PAR binding pocket in *purple*. The WWE domain of TRIP12 bound to ADP (*red*, 7UW7). The DTC domain of DTX2 bound to ADPr (*green*, 6Y3J (140)). See also the macrodomain fold in panel *E* that is sometimes used to bind PAR. *E*, two notable PAR erasers during the DNA damage response; PARG catalytic domain bound to ADPr (*dark teal*, 4B1H (155)) and ARH3 bound to ADPr (*green*, 6D36 (156)). ADPr, ADP-ribose; ARH3, (ADP-ribosyl)hydrolase 3; BAD, benzamide adenine dinucleotide; PAR, poly(ADP-ribose); PARG, poly(ADP-ribose) glycohydrolase; PARP, poly(ADP-ribose) polymerase.

ADP-ribose is an ancient protein and nucleic acid modification that has been utilized in many organisms, often as a defense mechanism (4). Mammalian cells employ ADP-ribose modifications in a variety of cellular contexts, including antiviral defense/innate immunity, protein homeostasis, gene regulation, and DNA repair/genome maintenance (5). Notably, in addition to single ADP-ribose (ADPr) unit modifications, multiple ADPr can be joined in a polymer known as poly(ADP-ribose) or PAR. PAR chains can be linearly elongated through the formation of a (2′-1″) ribose–ribose glycosidic bond between ADPr units. Occasionally, a (2″-1″) ribose–ribose bond can occur which branches the polymer (Fig. 1*A*) (6, 7). Although the majority of published studies have investigated ADPr modification of proteins, there is growing evidence and appreciation of the prevalence and importance of ADPr modification of nucleic acids (8, 9, 10).

This review highlights our current understanding of the human enzymes employed in ADPr modification catalysis, turnover, and signaling, with a focus on genome maintenance and poly(ADP-ribose) polymerase (PARP) enzymes. PARP inhibitors (PARPi) are important tools for understanding the biology of ADPr signaling, and several PARPi are approved for use in cancer treatments. The review also covers our current knowledge on PARPi mode of action, with a particular focus on clarifying the enigmatic process known as PARP “trapping.”

### Enzymes regulating ADPr signaling (writers, readers, erasers)

#### Writers

ADPr modifications are catalyzed by ADP-ribosyltransferase (ART) enzymes that take an ADPr group from NAD^+^ and attach it to macromolecules. Proteins can be modified on a variety of amino acid sidechains, including Glu, Asp, Ser, Arg, and Cys (5). Nucleic acids can receive the ADPr modification on phosphorylated termini and on nucleobases (8, 9). The ADP-ribosyltransferase diphtheria toxin-like family, containing the mammalian PARP enzymes, is defined as enzymes carrying an H-Y-[E/D/Q] signature motif in their NAD^+^ binding sites (5) (Fig. 1*B*). More specifically, their active site is composed of a “donor” site split into a nicotinamide binding pocket, in which the signature catalytic triad is located, and an adenine binding pocket (7). The “donor” site effectively holds the ADPr moiety that will be attached to either a target protein/nucleic acid or a PAR chain undergoing elongation. PAR chain elongation also requires the presence of an “acceptor” site pocket that holds the ADPr moiety, already attached to its target, to which a new ADPr unit from the “donor” site is added (7). As most members of the ADP-ribosyltransferase diphtheria toxin-like family do not catalyze PARylation, they also do not possess such “acceptor” sites. PARP enzymes involved in genome maintenance that can catalyze the formation of PAR chains include PARP1, PARP2, TNKS1 (PARP5a), and TNKS2 (PARP5b) (Fig. 1*C*). PARP3 also participates in DNA repair but catalyzes the addition of a single unit of ADPr, termed mono-ADP-ribosylation (MARylation). A later section will discuss some of the mechanisms regulating the writers and their specific roles in genome maintenance.

#### Readers

ADPr readers are comprised of a variety of binding modules that recognize PAR or MAR without removing the modification. Many DNA repair enzymes possess such modules as they are recruited to the site of damage *via* PAR. Among the high-affinity binding modules are the PAR-binding motif (11) and the PAR-binding zinc fingers (PBZs) (12). For example, while p53 (a transcription activator) and XPA (a scaffolding protein involved in nucleotide excision repair) bind PAR through a conserved PAR-binding motif motif (13), the histone chaperone aprataxin and polynucleotide kinase like factor (APLF) carries two PBZ motifs (12). The tandem PBZ motifs of APLF were found to recognize PARP2 branching (6), although it is currently unclear how they may coordinate to mediate such binding (14). In fact, APLF preference for PAR branches could not be reproduced in a recent study (15). PAR branching is generally accepted to be of low abundance, which could explain the difficulty in identifying modules specifically recognizing this modification. Other PAR-binding modules include WWE domains and BRCT domains (Fig. 1*D*) (13). Of note, RNA- and DNA-recognition motifs, like the oligonucleotide/oligosaccharide-binding fold, can also interact with PAR as it is essentially a nucleic acid polymer chemically similar to RNA and DNA. In fact, many readers carrying such modules will shift their interaction between PAR, RNA, and DNA, depending on the PAR chain length (13). PAR readers involved in the DNA damage response (DDR) are further discussed below.

#### Erasers

Enzymes that digest or remove ADPr modifications are referred to as erasers. Notable PAR erasers during the cellular response to DNA damage include poly(ADP-ribose) glycohydrolase (PARG) and (ADP-ribosyl)hydrolase 3 (ARH3) (Fig. 1*E*). Many thorough reviews have recently been written about PARG, and ARH3 with a focus on structure, substrate recognition, and function (16, 17). We provide a summary of their activities in this section. PARG hydrolyzes with high efficacy the ribose–ribose bonds within PAR chains. As such, PARG degrades linear and branched chains, but cannot remove the last, protein-linked moiety of ADPr, thus leaving a MARylation mark on its targets (18, 19, 20). Interestingly, PARG acts both as an *exo*-glycohydrolase (degrading PAR starting from its terminus, releasing ADPr units) (21), but also has a weak *endo*-glycohydrolase that releases PAR fragments (longer than three ADPr units) that are subsequently degraded further by PARG itself, albeit inefficiently (20, 22). The removal of the MARylation left by PARG is catalyzed by the action of mono-ADP-ribosyl-acceptor hydrolases. ARH3 is one such hydrolase acting during the DDR that removes serine-linked ADP-ribosylation in both MAR and PAR forms (23). Erasers capable of removing MARylation from Glu/Asp residues are typically macrodomains, such as MacroD1, MacroD2, and terminal ADP-ribose glycohydrolase 1 (24). Of note, many erasers that remove ADPr modifications on proteins can also remove this modification on nucleic acids. For example, phosphate-linked DNA and RNA MARylation can be reversed by PARG, MacroD2, terminal ADP-ribose glycohydrolase 1, and ARH3 (9), and adenine-linked PARylation can be removed by PARG (8).

### PARP enzyme regulation

There is still much work to do to establish the regulatory mechanisms of PARP family enzymes. However, recent work has elucidated key aspects of how PARP1 activity is regulated through interaction with DNA strand breaks, which is the most potent stimulator of PAR production in cells. Indeed, PARP1 is the most abundant PARP enzyme and the primary PAR writer in the cell, as its catalytic output accounts for approximately 80 to 90% of the PAR produced (25). PARP1 domain architecture is comprised of six independently folded domains: three zinc fingers (Zn1, Zn2, and Zn3), a WGR (Trp-Gly-Arg) domain, a BRCT domain and a catalytic (CAT) domain. The CAT domain is composed of the helical domain (HD) and an ART domain in which the active site is located (Fig. 1*B*).

**Figure 2:**
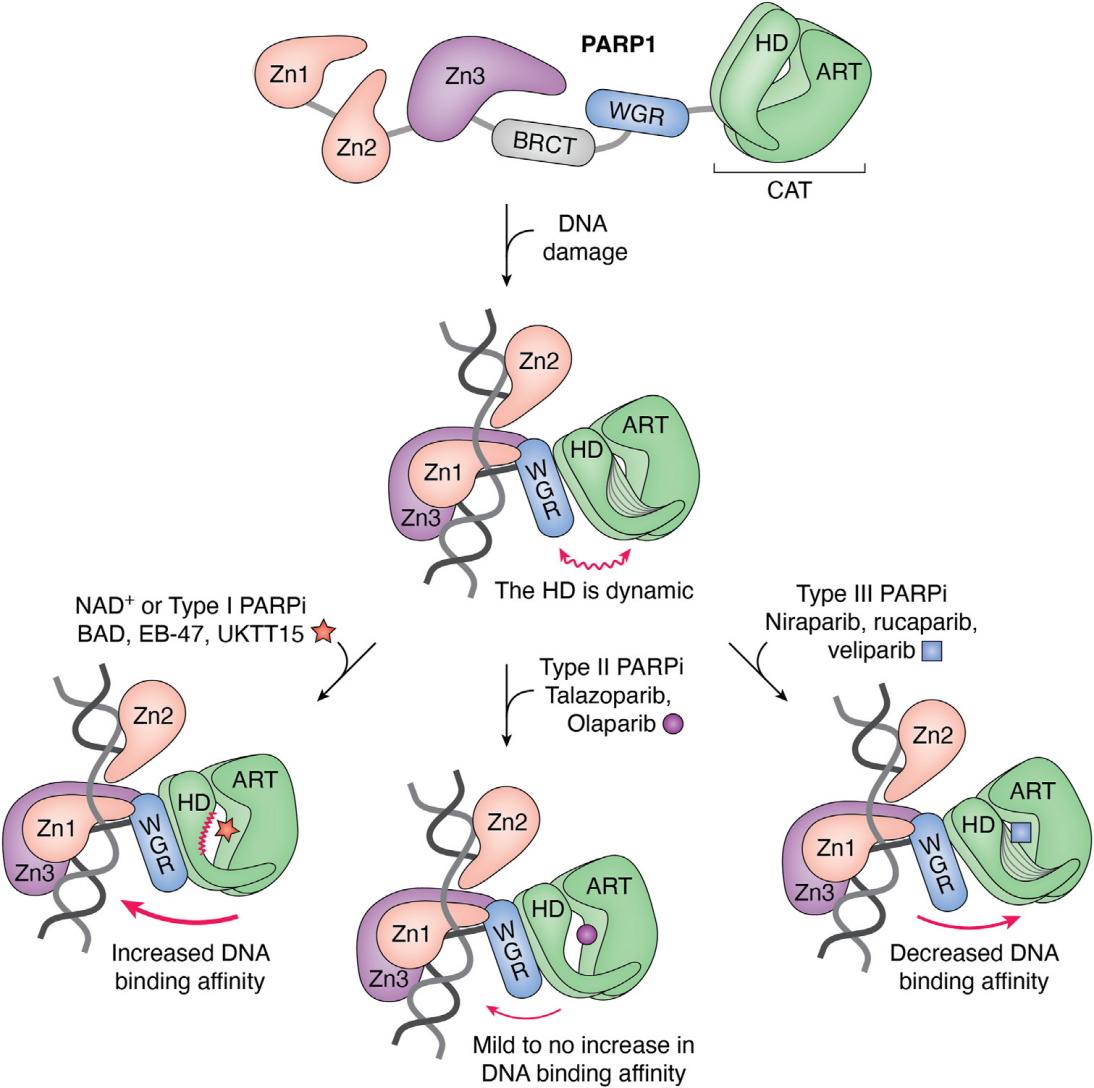
**PARP1 allostery and the impact of small-molecule interactions with the active site.***Top*, PARP1 domains behave as beads on a string in the absence of DNA damage, with HD closing the catalytic pocket of the ART domain to exclude NAD^+^. *Middle*, PARP1 domains collectively bind to DNA damage, which allosterically renders the HD into a dynamic state and thereby reveals the catalytic site of the enzyme to NAD^+^. *Bottom left*, binding of substrate NAD^+^, or type I PARPi, increases PARP1 DNA-binding affinity. *Bottom middle*, binding of type II PARPi mildly increases DNA-binding affinity or has no effect, while type III PARPi (*bottom right*) decrease PARP1 affinity for DNA damage. ART, ADP-ribosyltransferase; HD, helical domain; PARP, poly(ADP-ribose) polymerase; PARPi, PARP inhibitor.

PARP1 localizes to the nucleus where it scans intact chromatin *via* intrastrand transfer, also termed a monkey-bar mechanism (26). PARP1 intrastrand transfer requires the cooperative action of the three zinc fingers, the WGR and the BRCT domains to move from one DNA molecule to another (26, 27). PARP1 scanning of intact chromatin does not trigger its catalytic activity (27, 28). Rather, PARP1 is activated following the efficient organization of the zinc fingers and the WGR domain on the damage site (29, 30, 31), which relays an activating signal to the CAT domain. This allosteric communication opens the HD, relieving its autoinhibitory action (32), and causes the formation of an additional WGR-HD interface with a concomitant concerted rotation of the ART domain (33) to reveal the active site (Fig. 2). Of note, PARP1 recognition of DNA damage is not sequence-dependent and allows for PARP1 to interact with a variety of DNA lesions, such as single-strand breaks (SSBs), double-strand breaks (DSBs), and even apurinic and apyrimidinic sites in which the integrity of the backbone in preserved (29, 30, 34). Interestingly, while the BRCT domain contributes to PARP1 scanning of intact chromatin, it does not appear to be involved in DNA damage binding (27). On its own, catalytically active PARP1 primarily modifies aspartate and glutamate residues in the so-called “automodification region” comprised of the BRCT fold and a nearby linker region (35). PARP1 also modifies in *trans* other target proteins.

During the DDR, PARP1 undergoes a change of specificity as it collaborates with its cofactor histone PARylation factor 1 (HPF1) to modify serine residues on histones and itself (36). The newfound ability of PARP1 to modify Ser residues is due to the formation of a joint active site with HPF1, an interaction greatly favored by HD opening, in which HPF1 inserts a Glu residue in the catalytic pocket to deprotonate the acceptor Ser and initiate the ADP-ribosylation reaction (37, 38) (Fig. 1*B*). HPF1, being much less abundant than PARP1 in the cell, relies on a “hit and run” mechanism to form the joint active site at substochiometric ratios (39). Despite this short-lived interaction, HPF1 speeds up initial Ser modification events (39) and reduces PAR elongation as it sterically blocks the acceptor site (37). As such, Ser-linked PAR appears much shorter than Glu/Asp-linked PAR (39). Finally, HPF1 modulates PARP1 catalytic output by shifting the Ser-ADP-ribosylation balance toward histone modification relative to PARP1 automodification (39, 40), ultimately making Ser modification the most abundant modification during the DDR (41). Overall, this local burst in PAR initiates the DDR and recruits DNA repair factors that bind PAR (*i.e.*, readers). While PARP1 is steered toward histone modification in the presence of HPF1, it still automodifies itself on a triad of serine residues, namely S499, S507, and S519 (42). Mutating these serine residues was shown to retain PARP1 longer on DNA damage (42), suggesting that automodification is likely needed to trigger PARP1 timely release from damage during the repair process. PARylation being a highly negatively charged PTM, charge repulsion with nearby chromatin appears to be the driving force of the release (43, 44), although other mechanisms of enacting PARP1 release are still possible.

Another well-studied member of the PARP family that is activated by DNA damage is PARP2, the closest homolog of PARP1 (Fig. 1*C*). In contrast to PARP1, PARP2 only has a short, unstructured N-terminal region (NTR) and a WGR domain to accompany its CAT domain (45). Also, unlike PARP1, it is currently unclear how PARP2 navigates intact chromatin. However, PARP2 can be recruited to DNA damage *via* two mechanisms. PARP2 can either be recruited by binding the damage itself, an interaction mostly mediated by its WGR domain (45) that has specificity for 5′ phosphorylated DNA breaks (46). More specifically, PARP2 can interact with both the 5′ phosphate and the 3′ end of a DNA break, effectively “bridging” the broken strand or the DSB (47, 48, 49). Alternatively, PARP2 can be recruited to the site of damage using its NTR that binds PAR chains generated by PARP1 (6, 50). PARP2 is catalytically activated in both cases through a similar allosteric communication mechanism as PARP1 (32, 46, 51), making PARP2 a PAR writer as well as a reader (6, 45, 50). Upon activation, PARP2 appears to modify Glu/Asp residues with a higher ratio of branched chains than PARP1 and can add branched chains to preexisting PAR (6). During the DDR, PARP2 forms a joint active site with HPF1 in a manner very similar to PARP1 (37, 48, 49). This interaction has similar consequences for PARP2 catalytic output (*i.e.*, modification of Ser over Asp/Glu residues, faster initiation events but shorter polymers, transmodification over automodification) (36, 37, 39).

While PARP3 is also involved in the DDR, its specific role remains unclear. Similar to PARP2, PARP3 has an unstructured NTR, a WGR domain, and a CAT domain. PARP3 also appears to have high specificity for 5′ phosphorylated DNA breaks and is thought to be activated by a similar allosteric communication mechanism as PARP1/2 (46, 52). PARP3 MARylates Glu/Asp residues but does not form a joint active site with HPF1 (36, 39).

TNKS1 and TNKS2 (henceforth referred to as TNKS for simplicity) regulate a number of cellular functions including Wnt signaling, Golgi trafficking, and telomere length maintenance (53). They are also involved in SSB repair at telomeric sites of damage and DSB repair during homologous recombination (HR) and nonhomologous end joining (NHEJ) (53). In addition to the ART domain, TNKS harbor unique regulatory domains in the PARP family such as an N-terminal histidine, proline and serine-rich (HPS) domain (only present in TNKS1 and predicted to be unstructured), an ankyrin repeat domain, and a sterile alpha motif domain (54). TNKS do not catalyze the formation of branched PAR, but only of linear and somewhat small PAR chains of up to 20 ADPr units (55). They are also not autoinhibited as they do not carry a HD in contrast to PARP1/2/3 (Fig. 1*C*). Rather, their catalytic activity is positively regulated through self-polymerization, where their sterile alpha motif domain and ART domain engage in specific contacts that allosterically increase PARylation (56).

## ADPr contributions to genome stability and the cellular response to DNA damage

Perhaps the most studied function of ADPr in mammalian cells relates to its participation in genome maintenance. In this section, we highlight a few key roles of this modification during normal genome transactions and DDR and provide a few specific examples of factors that interact with the ADPr modification or are regulated by the modification.

### Chromatin remodeling during transcription and DDR

PARP1 and PARP2 are involved in the modulation of chromatin structure, not only during DDR but also during transcription. While PARP1 itself acts to condense chromatin and is primarily found within heterochromatin (57), PARylation is enriched at actively transcribed regions of the genome. PARP1 has been shown to form a complex with nucleosomes in the vicinity of promoters by outcompeting linker histone H1 (58). Subsequent PARylation of histones and other surrounding proteins allows for the recruitment of non-histone chromatin proteins HMGB1 and HMGB2, causing ATP-independent structural changes in the chromatin (59). PARylation simultaneously facilitates DNA uncoiling from the octamer and partial histone eviction. Taken together, these events promote transcription by lifting the nucleosomal barrier and permitting Pol II progression through the nucleosome (60).

PARylation at sites of DNA damage also mediates the recruitment of chromatin remodeling factors to reorganize the chromatin and facilitate lesion repair. Recruitment may happen *via* direct and/or indirect binding to PAR, highlighting that this process is deeply “layered.” During nucleotide excision repair, PARP1 catalytic activity at the nucleosome is increased by interaction with DNA damage–binding protein 2 (DDB2, previously known as XPE) (61). Ser-linked PARylation found both on PARP1 and histones is recognized by the chromatin-remodeling helicase ALC1, which repositions the nucleosome and exposes the site of damage for repair (61, 62, 63, 64). Interestingly, ALC1 recognition of PAR is mediated by its macrodomain fold that bears no catalytic eraser activity (62).

ALC1 also participates in the recruitment of the histone chaperone APLF during NHEJ (65). Alternatively, APLF may also be recruited *via* direct binding of ADP-ribosylated histones (66). While APLF facilitates DNA repair by displacing histones, it might also be responsible for the recruitment of specific histone variants during DNA repair, like macroH2A1.1 (67). Interestingly, macroH2A1.1 has been found to bind PAR, yet another example of the “layering” at play during chromatin remodeling and DDR (68). Other chromatin remodelers recruited in the NHEJ pathway *via* PAR binding include SMARCA5 and CHD5 (69). Interestingly, transcription repression complexes are also needed during DSB repair to pause transcription and allow correct repair. One such complex directly recruited through PAR binding is the nucleosome remodeling and deacetylase complex (70).

### Recruitment of DNA repair factors

Decompaction of chromatin allows for the recruitment of DNA repair factors to process the damage. Here, a few notable factors are enumerated. During SSB repair, XRCC1 is recruited to the site of damage *via* its central BRCT domain binding to PAR generated by both PARP1 and PARP2 (71) (Fig. 1*D*). XRCC1 is a scaffold protein, and it coordinates the formation of complexes with a variety of partner proteins to mediate the repair of nicks or gaps in the DNA backbone (72). For example, XRCC1 can form complexes with DNA polymerase beta, DNA ligase 3 (LIG3), polynucleotide kinase 3′ phosphatase, and aprataxin (72). In addition to PAR, XRCC1 also binds to DNA in a nonoverlapping pocket of its central BRCT domain, which could allow XRCC1 to remain at the damage site once PARP1 has been released (73). Studies indicate that XRCC1 and LIG3 can interact directly with PARP1 during SSB repair (74, 75); however, the impact of these potential protein–protein interactions on the efficacy of repair remains unclear, possibly because it could be overshadowed by the contribution of the ADPr-mediated interactions. XRCC1 can potentially directly interact with PARP1 *via* the heterodimerization of their respective BRCT domains (74, 76) although contradictory results were obtained on this possibility (77). Concerning LIG3 potential interaction with PARP1, it does not appear to be mediated by their respective BRCT domains (75, 76). During DSB repair, break sensing involves the recruitment of the protein kinase ATM and the DNA nuclease MRE11, both carrying PAR-binding modules. BRCA1 recruitment and PARylation stabilizes the BRCA1–RAP80 complex for efficient HR. Finally, PARP1 PARylates the catalytic subunit of DNA-dependent protein kinase, which stimulates its kinase activity and ultimately drives NHEJ (69). PARylation of BRCA1 and the catalytic subunit of DNA-dependent protein kinase represent two examples of the ADPr modification modulating the function of target proteins. Overall, the functional consequences of PAR binding by DNA repair proteins have been more studied than the functional consequences of the ADPr modification of DNA repair proteins. It appears plausible that the ADPr modification of DNA repair factors in some cases might not have an effect on their biochemical function, but rather simply contribute to their recruitment to or retention at sites of damage, for example, as discussed in the following section in the context of liquid–liquid phase separation or phase condensate formation.

### Phase condensates

In addition to mediating the recruitment of DNA repair factors, PAR can also trigger liquid–liquid phase separation in the vicinity of lesions to potentially favor repair. PAR thereby acts as a scaffold and creates a local environment suited for the enrichment of client proteins such as members of the FET family (FUS, EWSR1, and TAF15) (78). In a biochemical analysis of PAR structure, the inherent rigidity of PAR chains was shown to be counteracted by cations coordinating the phosphate negative charges, thereby causing an abrupt compaction of PAR favorable to condensate formation (79). Similarly, the presence of highly positively charged FUS triggers an equivalent rapid transition, which is not reproduced by the WWE reader domain of RNF146, highlighting that not all PAR-binding proteins may promote the formation of condensates (79). While FUS can also form condensates with DNA and RNA, it appears that FUS interaction with PAR, although transient, may prime FUS into forming stable condensates unaffected by subsequent PAR digestion, in stark contrast to FUS-RNA condensates that dissolve after treatment with RNase (80). Interestingly, inhibiting TNKS1 catalytic activity, but not PARP1, resulted in less PAR-seeded FUS condensates in this context (80). Other proteins enriched in PAR condensates require first binding to PAR to trigger their later PAR modification, like p53 (81), which raises the possibility that initial PAR binding events induce the formation of condensates dependent on both noncovalent and covalent PAR modification. It is not yet known how condensate composition may change if subjected to partial digestion by PAR erasers.

### Replication stress and unligated Okazaki fragments

PARP1 appears to be involved in both the regulation of the speed of replication fork movement and the management of defective replication forks. Replication forks moving at speeds above a certain threshold accumulate DNA damage, ultimately compromising genome integrity. Loss or inhibition of PARP1 was shown to accelerate forks, highlighting that PARP1 recruitment and catalytic output plays a role in modulating fork progression speed (82). The recruitment of PARP1 to replication forks is facilitated by CARM1, an arginine methyltransferase, but the recruitment occurs independent of CARM1 catalytic activity (82). PARP1 is involved in managing defective replication forks *via* two mechanisms. First, in the presence of DNA damage at replication forks, PARP1 automodification and modification of p53, will cause p53 recruitment and activation of p21. Simultaneously, PARP1 automodification releases p21 from p21–PARP1 complexes. Taken together, these events cause the accumulation of p21 and subsequent stalling of defective forks (83, 84). Second, recruitment of PARP1, HPF1, and CARM1 as a complex increases PAR production to promote fork reversal. RECQ1 binding to PAR inhibits its helicase activity, thereby preventing stalled fork restart (82), and allowing for fork reversal driven by the recruitment of DNA translocases (85), thus favoring high fidelity replication over speed and damage tolerance.

PAR contribution to genome stability involves its participation in the processing of unligated Okazaki fragments in an endogenous context (*i.e.*, a context in which cells have not been exposed to DNA damaging agents like methyl methanesulfonate) (86). In this case, PARylation found at replication forks in S-phase is not triggered by replication stress, but by the presence of unligated Okazaki fragments that have eluded the canonical FEN1 and LIG1 pathway of repair. PARP1 and PARP2 act as sensors, generating PAR to which the BRCT domain of XRCC1 binds, ultimately promoting Okazaki fragment processing (86).

### Stress signaling and parthanatos

PARP1 overactivation during catastrophic DNA damage accumulation can lead to cell death owing to a mechanism that is still under scrutiny. It has been suggested that substrate NAD^+^ depletion upon intense PARP1 activation may be the main cause of death, specifically in neurons, as NAD^+^ is an important cofactor in many redox reactions (87). However, others have shown that while the levels of cytosolic and nuclear NAD^+^ are greatly diminished following PARP1 overactivation, mitochondrial NAD^+^ pools remain largely unchanged and can partially rescue cell death, suggesting that NAD^+^ depletion alone is not sufficient for cell death (88). Rather, it appears that production and release of free PAR participates in cell death in a caspase-independent pathway termed parthanatos. Migration of PAR chains from the nucleus to the mitochondria induces the release of apoptosis inducing factor (AIF) into the cytoplasm (89), an event possibly triggered by AIF binding to PAR (88). AIF then forms a complex with the macrophage migration inhibitory factor, and this complex in turn translocates to the nucleus where it causes large-scale DNA fragmentation and ultimately cell death (90). Interestingly, it was found that PARG KO cells are especially sensitive to PAR accumulation and that ARH3 does not rescue cells from parthanatos (91). During parthanatos, PAR chains longer than 60 ADPr units appear the most toxic; however it is unclear how such long PAR chains can evade digestion by PARG (88).

### Ser-linked ADPr: A two-speed signaling modification

**Figure 3:**
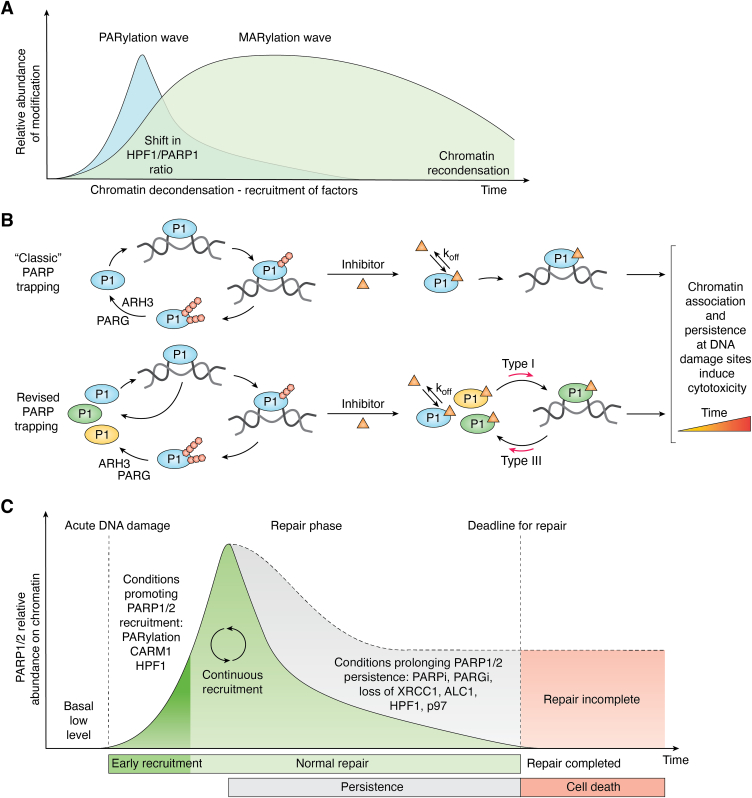
**ADPr signaling waves and PARP1/2 trapping on DNA damage.***A*, Ser-linked ADP-ribosylation during DNA damage functions as a two-speed mechanism with an early and short-lived sharp increase in PARylation, followed by a delayed but long-lasting wave of MARylation. *B*, *top*, the “classic” interpretation of PARP trapping. PARP1 is recruited to DNA damage and automodifies itself. PARylation causes PARP1 to release from damage, in which case digesting the modification with PARG and ARH3 allows PARP1 to be recycled to the lesion until the repair it completed. In the presence of an inhibitor (*orange triangle*), PARP1 stalls on DNA damage as it cannot automodify itself, leading to PARP1 being observed on chromatin. The binding kinetics of the inhibitor, in particular the inhibitor off-rate (k_off_), strongly impact the amount of residual PAR that is produced. *B*, *bottom*, the revised interpretation of PARP trapping. The population of PARP1 molecules in the cell cycles on and off the break. The bound PARP1 molecule undergoes PARylation and falls off, in which case another PARP1 molecule is recruited (continuous recruitment). In the presence of an inhibitor, PARP1 molecules continue to exchange on the DNA break; however, it may be retained longer, especially in the context of a type I inhibitor. On the other hand, type III inhibitors shift the population toward a shorter persistence on damage. Taken together, the residency time of the inhibitor in the active site (k_off_) and the “allosteric type” of the inhibitor govern PARP1 overall persistence on DNA damage. *C*, acute DNA damage triggers PARP1/2 recruitment to DNA damage. The early recruitment is facilitated by the presence of histone PARylation factor 1 and CARM1. Initial PARylation may also in turn favor the early recruitment of additional PARP1/2 molecules. The repair phase will proceed with PARP1/2 PARylating themselves and falling off which will allow other PARP1/2 molecules to interact with DNA damage (continuous recruitment) until the repair is completed. Conditions prolonging PARP1/2 persistence on damage may delay the repair until a deadline is reached. In such case, the high amount of DNA damage left is lethal to cells. ADPr, ADP-ribose; ARH3, (ADP-ribosyl)hydrolase 3; MARylation, mono-ADP-ribosylation; PARP, poly(ADP-ribose) polymerase.

It has recently become increasingly evident that the two forms of Ser-linked ADP-ribosylation (*i.e.*, MARylation and PARylation) coexist during DDR and that MARylation found at lesions is not simply a by-product of PAR chain degradation (92). More specifically, while an intense short-lived PAR signaling wave rapidly forms at sites of lesions, a recent study highlighted that a delayed, yet long-lived, MAR signaling wave will take over, even after PAR removal (93) (Fig. 3*A*). This two-phase signaling wave appears to be caused by a shift in the PARP1/HPF1 ratio, HPF1 dissipating from the sites of damage slower than PARP1 (66, 93), which increases the probability of making MARylation due to the HPF1 block on PAR formation (37). As MARylation facilitates chromatin decompaction and the recruitment of certain repair factors (66), but does not seem as toxic as PARylation over time (94), it seems that it represents a safer option to effectively carry out long lasting ADPr-mediated signaling. These recent findings also raise the likelihood that mono-ADPr readers will play an important role in regulating the second wave (93).

## Consequences of inhibiting ADPr signaling

### PARP inhibition in the context of cancer treatment

Four inhibitors of PARP (olaparib, talazoparib, rucaparib, and niraparib) have been approved for the treatment of cancers associated with *BRCA1* and *BRCA2* mutations (95) as PARPi were found to be specifically lethal to BRCA1/2-deficient cells, following the principle of synthetic lethality (96, 97). Synthetic lethality is a term used to describe the fact that neither BRCA1/2 deficiency nor PARPi are lethal individually, but the combination of PARPi and BRCA1/2 deficiency is cytotoxic. Synthetic lethality caused by PARPi treatment has been attributed to an accumulation of SSBs degenerating into DSBs during replication that cannot be repaired since BRCA1/2-deficient cells are HR defective (96, 97). However, many studies have shown that DSBs do not accumulate in WT or BRCA-defective cells treated with PARPi, contrary to what is expected (98). Alternatively, an accumulation of gaps during replication has been proposed to be the main culprit of synthetic lethality (99). Briefly, an increase in the speed of replication fork movement following PARPi exposure causes fork lengthening (83), which in turn is associated with the formation of gaps that do not degenerate into DSBs. Since PARP1 is known to participate in Okazaki fragment processing (86), these gaps could in fact be unligated Okazaki fragments. While it is unclear whether synthetic lethality arises from the number of gaps or their persistence, cells that display replication gap suppression become resistant to PARPi treatment (100). Additionally, PARPi were found to sensitize cells in which fork protection was compromised, but HR was not necessarily compromised. Taken together, these results highlight that replication gaps likely play a key role in PARPi-induced synthetic lethality.

### The complex relationship between PARPi and PARP trapping

PARPi generally carry a nicotinamide/benzamide pharmacophore designed to mimic the interaction that substrate NAD^+^ makes with highly conserved residues of the active site (101). PARPi therefore hinder the formation of PAR chains as they are competitive inhibitors of NAD^+^ binding. As outlined above, autoPARylation participates in PARP1 release from DNA damage, possibly due to charge repulsion with nearby chromatin (43, 44), which entails that PARPi should have the capacity to prolong PARP1 association with chromatin. Accordingly, the term PARP “trapping” was first introduced to describe the observed enrichment of PARP1 in the chromatin-bound fraction upon PARPi treatment (43, 102). More recently, fluorescence microscopy combined with a pre-extraction step to detect chromatin-bound proteins has been used to quantify trapping of newly developed PARPi that are being evaluated in clinical trials, namely AZD5305 and AZD9574 (103).

The concept of PARP trapping remains poorly characterized as this phenomenon appears more complex than originally anticipated. The main cause of trapping over the years has been attributed to catalytic inhibition, as the lack of automodification was shown to hinder PARP1 release (42). Interestingly, inhibitors can also impact PARP1/2 affinity for DNA damage by modulating enzyme allostery (104) (Fig. 2). A nonhydrolysable mimic of substrate NAD^+^, benzamide adenine dinucleotide (BAD), was found to stabilize preexisting interactions with DNA damage, thereby strengthening DNA binding and causing DNA retention *in vitro* (52). A classification of PARPi based on their propensity to influence PARP1 allostery classified BAD, along with EB-47 and UKTT15 (a veliparib analog), as type I inhibitors causing increased retention on DNA breaks (104). Type II inhibitors were found to show mild (talazoparib) to no (olaparib) retention capability. Niraparib, rucaparib, and veliparib were found to belong to a class referred to as type III inhibitors that actually promote PARP1 release from DNA damage. Recent studies have highlighted that trapping potency may in fact be largely dictated by PARPi residency time in the active site (*i.e.*, off-rate) (105), which is dictated by the way inhibitors engage the active site (101). For example, inhibitors displaying a high off-rate (veliparib and niraparib) are more prone to release from the active site and to allow residual PARylation, while inhibitors with a low off-rate (talazoparib and olaparib) may not (106). Interestingly, this classification also somewhat corresponds to their “allosteric type” as measured *in vitro* in the absence of NAD^+^ (107). While these off-rates were measured in the absence of HPF1, a recent study has highlighted that HPF1 can potentially modulate PARPi residency time (108). In fact, it appears that inhibitor potency might correlate better with the off-rate measured for the PARP1–HPF1 complex rather than PARP1 alone (108). This finding has important implications since PARPi are not typically designed taking into account the contribution that HPF1 makes to the PARP1 and PARP2 active site (49). Overall, PARP trapping appears to be induced *via* two main causes: first, catalytic potency with PARPi residency time being a key parameter, and second, PARPi modulation of allostery and DNA-binding affinity.

One factor that complicates our understanding of PARP trapping is that the definition of the phenomenon itself is rather vague. Historically, PARP1 trapping has been described as the prolonged binding of PARP1 at sites of damage, yet the name “trapping” suggests a complete physical stalling of the enzyme for a seemingly infinite amount of time. Since measurements of chromatin-bound proteins only represent a snapshot of binding events at a specific given time, it remains unclear how long PARP1 binding to DNA should last to qualify as trapping. Many recent publications have sought to better characterize the kinetics of PARP1 recruitment to DNA damage in the presence of an inhibitor to better define this phenomenon.

Quantitative live-cell imaging showed that PARP1 persisted longer at sites of laser-induced DNA damage in the presence of talazoparib and niraparib (109). However, fluorescence recovery after photobleaching (FRAP) revealed that PARP1 persistence was the result of multiple PARP1 molecules being continuously recruited to the site of damage (109), in stark contrast to what is expected for a completely stalled enzyme on chromatin. As the FRAP assay cannot measure the kinetics of individual PARP1 molecules at breaks, live-cell single-molecule microscopy offers a simplified system to deconstruct these binding events (110). PARP1 molecules exposed to breaks during imaging were shown to continue to diffuse freely or to engage in binding events, either stably or transiently. Treatment with talazoparib converted transiently bound PARP1 molecules into stably bound molecules and increased their dwell time at sites of DNA damage (110), while treatment with olaparib showed no difference in the behavior of the molecules. Of note, the impact of type I inhibitors on PARP1 behavior was not studied with this assay. *In vitro* single-molecule assays of PARP1 and DNA colocalization, conducted in the absence of NAD^+^, showed that type I inhibitors noticeably increased the fraction of molecules displaying persistent binding, while type III inhibitors favored an intermittent-binding behavior, consistent with their “allosteric type” (107). In the presence of NAD^+^, type I inhibitors still promoted persistent binding relative to the other types, albeit more modestly (107), likely reflecting that PAR production is the more potent regulator of PARP1 release from DNA. Interestingly, PARPi effect on DNA binding in this context overall appeared in line with the inhibitor residency time or off-rate (106, 111). Taken together, these results highlight that PARP trapping cannot simply be described as a complete physical stalling of the enzyme on damage.

The contribution of allostery to PARP trapping has been the subject of debate across the years. The study of allosteric communication within PARP1 made a leap forward following the identification and *in vitro* characterization of substrate analog BAD, which showed that catalytic pocket occupancy can promote PARP1 retention on a DNA break (52). The subsequent classification of inhibitors in three “types” as described in a previous section (*i.e.*, type I, II, III) (104), highlighted that inhibitors may modulate PARP1 retention in different ways. The current toolset of type I inhibitors is underdeveloped, thus the analysis of their effect on PARP1 allostery in cells is limited at this point. However, details on the contributions of PARP1 allostery to trapping may be derived from the analysis of specific PARP1 mutations. For example, the retention of catalytically deficient PARP1 mutant E988A during single-molecule colocalization assays was decreased in the presence of talazoparib and olaparib, in direct contrast to WT PARP1 (107). In biochemical assays, a mutant disrupting PARP1 allosteric communication (Zn3-WGR-HD interface mutant W318R) could not be retained at sites of damage in the presence of the type I inhibitor EB-47, illustrating a defective allosteric communication that cannot convey allostery properly across PARP1 domains (107). Interestingly, a PARP1 mutant (ΔVE; deletion of two residues in the HD) known to highly promote a proretention state *in vitro*, was found to persist at sites of damage produced during microirradiation assays in cells (33). This mutant exhibits elevated levels of PAR production, yet the increased abundance of ADPr modification does not appear to promote release from DNA. It is thus likely that the observed persistence at DNA lesions reflects its perturbed allosteric communication that increases DNA-binding affinity (33). Together, these results with PARP1 mutants emphasize that allostery is an inherent property of PARP1 structure that most likely contributes to the puzzle of PARP trapping.

While most studies on PARPi have used PARP1 to test their impact, inhibitors targeting PARP1 are often promiscuous to other PARP family members, including PARP2, as they share a similar ART domain (5). A recent study has shown that PARPi may impact PARP2 allostery to a different degree and with different outcomes. Niraparib, talazoparib, rucaparib, and olaparib were all found to classify as type I inhibitors *in vitro* since they greatly strengthened PARP2 DNA binding, while veliparib reduced DNA binding and thus showed type III inhibitor behavior (112). Structural differences in helices of the HD in PARP1 and PARP2 could explain these differences and highlight how the HD acts as a sensor for small molecules bound to the PARP1/2 active site (112). While PARP1 foci recovery during FRAP assays is largely unchanged in the presence of niraparib and talazoparib (109), PARP2 foci recovery is significantly delayed (50, 113), to an extent that almost resembles an actual physical stalling of the enzyme on DNA damage. Interestingly, the FRAP recovery of a catalytically deficient PARP2 mutant (E545A) was similar to WT, suggesting that the recovery delay cannot be attributed to catalytic inhibition alone (50). In light of these differences, further work is required to better characterized PARP2 persistence at sites of damage with single-molecule techniques.

Although PARP trapping is not generally considered to result from a covalent attachment of PARP1 to DNA damage, a covalent bond may form during base excision repair (BER) as PARP1 possesses a weak apurinic/apyrimidinic lyase activity capable of incising the site of a lesion. The incision creates a single-nucleotide gap ready for further processing (34); however, the incision may stall, resulting in the formation of a DNA-protein cross-link (DPC) (114). In this context, the primary residues targeted for cross-linking are cysteine residues found in the PARP1 Zn1, Zn3, and BRCT domains (115). The PARP1-DPC appears to be sufficiently long-lived (114) to require a subpathway of BER to be resolved in a replication-independent context (116). Despite apurinic/apyrimidinic sites being considered very abundant lesions (117), and studies identifying a higher level of PARP1-DPCs in cells following PARPi exposure and methyl methanesulfonate treatment (118), a PARP1 covalent link to DNA damage remains poorly characterized, therefore precluding the evaluation of the contribution of covalently linked PARP1 to the trapping phenomenon. Of note, PARP1 participates in the repair of DPCs of topoisomerase 1, possibly *via* a direct protein–protein interaction with phosphodiesterases TDP1 and TDP2, which hydrolyze the covalent bond of the DPC (119).

In light of these recent results, we propose an evolution of the PARP trapping model (Fig. 3*B*). The current evidence indicates that PARP trapping should not be considered a prolonged physical stalling of PARP1 and/or PARP2 at the sites of damage, but rather the continuous recruitment and persistence of PARP molecules. This continuous recruitment arises by a shift in the population behavior of molecules. Molecules displaying intermittent binding switch to a stably bound state, resulting in an increased fraction of the total population exhibiting a longer dwell time on DNA damage. Of note, the presence of a saturating amount of inhibitor, regardless of its “allosteric type” does not preclude that most PARP1 molecules continue to diffuse freely and transiently bind. Although the term “trapping” has been useful in defining a general concept and will likely continue to be used, it is important that the recent mechanistic insights are understood, as it currently represents a desirable characteristic for therapeutic approaches. At the same time, one could ask: how long does PARP1 or PARP2 need to stably bind to DNA damage to negatively impact the repair process? Answering this question is tricky at best and we can only hypothesize by considering the relative consequences of PARP1/2 persistence on different repair pathways.

## Other factors modulating PARP1 retention on chromatin

Factors promoting PARP1 catalytic activation have the potential to modulate its retention on chromatin as autoPARylation participates in PARP1 release from damage (42). PARP1 was found to display increased catalytic activity in the presence of CARM1 (82), TSG101 (120), SpinDoc (121), HMGB3 (122), and HMGA2 (123), to name a few. With the growing number of factors that seemingly increase PARP1 catalytic activity (124), it is important to remember that little is known about how PARP1 interacts with these factors, in contrast to HPF1, and how they may impact PARP1 allosteric communication.

The action of PAR erasers can also modulate PARP1 retention. For example, loss of PARG and the ability to remove PAR causes resistance to the PARP1-trapping effects of PARPi. In this scenario, it is expected that PARPi do not entirely prevent the production of PAR and that these residual amounts of PAR that would normally be reversed by PARG are restored and sufficient to modulate the persistence of PARP1 on DNA damage (125). Thus, PARG loss is able to promote PARP1 release, even in the presence of PARPi. Curiously, the inhibition of PARG can lead to the reverse consequence (*i.e.*, increased PARP trapping), owing to a mechanism that is still poorly understood (126). However, we hypothesize that this apparent trapping may be the result of an increased continuous recruitment of PARP1 to the site of damage as PARylation facilitates this process (127).

PARP1 retention on chromatin can also be modulated by factors competing for the site of damage. For example, in an endogenous context, XRCC1 was found to prevent the continuous recruitment of PARP1 at SSBs during BER by mediating the assembly of the polymerase beta/LIG3 complex to outcompete PARP1 at the site of damage (128). In the absence of XRCC1, robust PARP1 retention was accompanied by NAD^+^ depletion, suggesting that PARP1 continuous recruitment and digestion of PAR chains by erasers leads to loss of automodification over time, explaining PARP1 entrapment in this context (128).

How cells specifically deal with PARP1 trapped at sites of damage in the presence of an inhibitor has been a long-standing question in the field. Even now, it is unclear what proteins or factors might be recruited by a trapped PARP1 molecule, and what type of complex they may form together. Here, we highlight a few recent studies that aimed to characterize how trapped PARP1 can be removed from the site of damage.

As mentioned above, ALC1 is a chromatin-remodeling helicase whose recruitment is mediated by Ser-linked PARylation and triggers the repositioning of the nucleosome to expose the site of damage. ALC1 is thought to be involved in the removal of trapped PARP1 potentially by peeling off the stalled enzyme, following nearby chromatin remodeling (129). However, we are faced with a conundrum since contradictory results have shown that ALC1 only removes trapped PARP2, again potentially through a similar mechanism (113). While the true mode of ALC1 action remains unclear, the scientific community has reached a consensus that loss of ALC1 severely sensitizes HR-deficient cells to PARPi treatments, which represents a potential avenue to counter resistance in patients (113, 129, 130, 131).

Trapped PARP1 can be targeted for disassembly *via* a collaboration between two different PTMs, namely SUMOylation and ubiquitylation. Briefly, PIAS4 SUMOylates PARP1 which is then ubiquitylated by RNF4, making PARP1 a target for p97, an ATPase with a segregase/unfoldase activity (132). As such, p97 removes ubiquitylated substrates from chromatin by unfolding and disassembling its targets through its central pore (133). This process does not appear to be specific to PARP1 as p97 is also known to remove aborted topoisomerase 1 cleavage complexes from chromatin, among other substrates (134). Inhibiting p97 is lethal to BRCA1/2-deficient cells, highlighting that p97 inhibition can potentiate the effects of PARPi in human tumor cells (132). It appears that the timely removal of PARP1 through ubiquitylation can be modulated by the recruitment of ATXN3, which is SUMOylation and PARylation dependent (135). As a deubiquitylating enzyme, ATXN3 effectively breaks down ubiquitylation under normal circumstances to prevent premature disassembly of PARP1 from the site of damage (135).

Ubiquitylation alone can also modulate PARP1 retention on chromatin by triggering PARP1 proteasomal degradation. E3 ligases known as PAR-targeted ubiquitin ligases carry a PAR-binding module to mediate their recruitment to PARylated substrates. For example, both RNF146 (136) and TRIP12 (137) possess a WWE domain recognizing iso-ADPr (Fig. 1*D*), and CHFR possesses a PBZ motif (138). TRIP12 depletion was found to greatly sensitise BRCA-proficient cells to the PARPi olaparib (137). A few members of the DELTEX family of ubiquitin ligases, namely DTX1, DTX2, and DTX4, also carry two consecutive WWE domains (139). Interestingly, they were also found to possess a DTC domain that crystallized in complex with ADPr (Fig. 1*D*) and appears crucial for their catalytic activity (140). More specifically, the DTC domain acts as a mono-ADPr–binding module to position ADPr for modification with ubiquitin (141). It appears that the DTC domain also provides residues that contribute to catalyzing this unusual PTM (ADPr-ubiquitin) that was first observed *in vitro* (141).

The resolution of PARP1 trapped at DNA replication forks is carried out by SPRTN, a metalloprotease that degrades DPCs, leaving small peptide adducts on DNA that can be bypassed by the translesion synthesis pathway (142). In this specific context, trapped PARP1 is not crosslinked to DNA, yet it still triggers SPRTN-mediated degradation. SPRTN recruitment to both types of trapped complexes potentially requires its interaction with both DNA (143) and PARP1 (142). Another factor, namely FAM111A, was also found to digest PARP1–DNA trapped complex *via* its trypsin-like domain and therefore to carry a similar role as SPRTN (144). Interestingly, SPRTN may also participate in the degradation of an actual PARP1-DPC in a replication-dependent context in which the polymerase has extended the nascent DNA (143).

Taken together, these findings can be visualized in a schematic of PARP1/2 relative abundance on chromatin during DDR considering conditions that may influence their persistence or turnover (Fig. 3*C*).

## Conclusion and perspectives

PARP inhibitors have been a powerful tool to understand ADPr signaling, and the recent development of PARG inhibitors extends the toolset. Since the PARP family share a similar active site, PARPi designed to outcompete substrate NAD^+^ have been historically nonspecific. Engineering highly selective PARPi has been a long-term goal in the field to tackle this promiscuity. The recent development of AZD5305, an inhibitor highly selective toward PARP1, has shown promising results in preclinical trials (145). Interestingly, AZD5305 is deemed a potent PARP1 trapper, based on the detection of chromatin-bound proteins (145). *In vitro* biochemical data revealed that AZD5305 is likely a type II PARPi, as it does not appear to modulate PARP1 retention to DNA break (112). AZD5305 therefore represents yet another example of an inhibitor where trapping propensity is best represented by considering all facets that may induce the retention of the enzyme on damage. Designing inhibitors that take into account the revised PARP1 trapping model may yield inhibitors that are tuned to the desired trapping propensity and therefore the appropriate therapeutic outcome, especially as PARPi could potentially be used in the future to treat inflammatory and neurogenerative diseases (146, 147).

Despite the critical role for ADP-ribosylation in DNA repair and ultimately cell survival, this PTM has been little studied over the years due to its chemical and structural complexity. The recent development of tools to identify ADP-ribosylation targets and factors recognizing the modification will help cover gaps in current knowledge (148). For example, the development of Nano luciferase–based split luciferase “PAR-trackers” to detect PAR production in live cells under physiological conditions has shed light on the dynamics of PAR accumulation during adipogenesis (149). Additionally, modular antibodies highly sensitive to MARylation have highlighted that this modification acts as a second wave of signaling during the DDR (93). These antibodies have additionally allowed the identification of yet another factor recruited to lesions, RNF114, a ubiquitin ligase specifically recognizing MARylation (93). There are also indications that we will learn about other PARP enzymes that make contributions to the DDR, in particular the PARPs that carry out MARylation and might have gone largely unnoticed in the wake of the powerful first PAR wave (150, 151). It can also be expected that we will learn more about the prevalence, functions, and consequences of ADPr modifications on nucleic acids, as this area of research is developing. We can therefore expect more insights to come for ADPr contributions to genome stability and chromosome dynamics.

## Conflict of interest

J. M. P. is a cofounder of Hysplex LLC with interests in PARPi development. É. R.-T. declares no conflict of interest with the contents of this article.
